# From AKI to CKD: Maladaptive Repair and the Underlying Mechanisms

**DOI:** 10.3390/ijms231810880

**Published:** 2022-09-17

**Authors:** Zhiwen Wang, Chun Zhang

**Affiliations:** Department of Nephrology, Union Hospital, Tongji Medical College, Huazhong University of Science and Technology, Wuhan 430022, China

**Keywords:** AKI, CKD, maladaptive repair, fibrosis, renal tubular epithelial cells

## Abstract

Acute kidney injury (AKI) is defined as a pathological condition in which the glomerular filtration rate decreases rapidly over a short period of time, resulting in changes in the physiological function and tissue structure of the kidney. An increasing amount of evidence indicates that there is an inseparable relationship between acute kidney injury and chronic kidney disease (CKD). With the progress in research in this area, researchers have found that the recovery of AKI may also result in the occurrence of CKD due to its own maladaptation and other potential mechanisms, which involve endothelial cell injury, inflammatory reactions, progression to fibrosis and other pathways that promote the progress of the disease. Based on these findings, this review summarizes the occurrence and potential mechanisms of maladaptive repair in the progression of AKI to CKD and explores possible treatment strategies in this process so as to provide a reference for the inhibition of the progression of AKI to CKD.

## 1. Acute Kidney Injury (AKI) and Chronic Kidney Disease (CKD)

AKI and CKD are the two main types of kidney disease [1,2]. With improvements in living standards, the incidence of renal diseases is increasing significantly [3]. It is reported that the number of pathological deaths caused by AKI was about 2 million worldwide in 2013 [4], and the number of patients who died of CKD reached 1.2 million in 2015 alone [5,6]. Additionally, according to the prediction of the World Health Organization, the mortality rate of CKD may even reach 14 per 100,000 people by 2030. This trend shows that the progress of AKI and CKD has caused a heavy burden on healthcare systems, both in China and worldwide [6]. Previous studies have suggested that AKI is a self-limited disease with a clear line dividing it from CKD. However, combined with the conclusions of recent clinical and basic studies, there is an urgent need to update the understanding of AKI. There is a close relationship between AKI and CKD. The existence of CKD is the promoting factor of AKI, and the occurrence of AKI is the factor that cannot be ignored in aggravating CKD [7,8]. Both of these are important factors leading to increased mortality or end-stage renal disease (ESRD), so it is of great significance to explore the relationship between them.

AKI is an acute clinical syndrome which can be caused by various factors such as nephrotoxic drugs, ischemia, severe infection, etc. Rapid decline in renal function, electrolyte disturbance and acid-base imbalance are considered typical symptoms of AKI. In ICUs, the probability of AKI leading to death is up to 50% [9,10]. The pathological progression of AKI can be divided into four levels: mild, self-limited, severe and persistent, which decide the degree of the adverse consequences [11]. Recently, CKD is increasingly regarded as a consequence of continuous progress of AKI [12]. Coca et al. reported that, after adjusting several important confounding variables, the risk of CKD, ESRD and other adverse consequences in patients with AKI “recovery” was significantly higher than that of patients without AKI, and this probability increased significantly with the increase in the severity of AKI [13]. In addition, the same results were also found by Kim et al. [14]. Ishani A et al. indicated that patients diagnosed with AKI had an eight-fold higher risk of developing ESRD compared with patients with no history of AKI or CKD after adjusting for factors such as age and sex [15]. From all these studies, we can see that the occurrence of AKI sooner or later increases the incidence of CKD and even ESRD to a great extent. As for the mechanism, the transformation of AKI to CKD is closely related to the complex interaction between the injury of the renal tubular epithelial cells, endothelial cell dysfunction, inflammatory progression and interstitial fibrosis. In the following sections, we elaborate on these in detail.

The existence of AKI promotes the occurrence of CKD. Similarly, CKD patients are also at high risk of AKI. An increasing number of retrospective studies have proved that the existence of CKD was indeed one of the most vital risk factors for the occurrence of AKI [16,17]. The reason may be that the decrease in the number of nephrons in patients with CKD is closely related to the deficiency of renal homeostasis under acute stress. James et al. showed that reduced eGFR increased the incidence of AKI to some extent in CKD patients with or without hypertension or diabetes [18]. Similarly, Ishani A et al. reported that their results showed that the incidence of AKI in patients with CKD was four times higher than that in non-CKD patients [15]. Accordingly, the increase in the incidence of AKI caused by the presence of CKD may be due to the delay in renal repair and recovery after AKI. In 2012, Zhou et al. showed that patients with pre-existing CKD had a significantly increased risk of acute renal function deterioration on admission, and only 30.7% of patients with CKD were able to recover their creatinine levels after acute AKI [19].

## 2. Adaptive and Maladaptive Repair after AKI

Previously, because of the self-repairing ability of the kidney, it was thought that AKI was widely considered as a self-limited disease. However, with the deepening of research and the knowledge gained from clinical experience, more and more attention has been paid to the potential late complications of AKI [20]. Abundant studies have confirmed that the best result of AKI is a complete recovery to normal structure and function. However, most patients face poor recovery instead of complete recovery [21]. The pathological process of AKI can be divided into three stages: the development stage, the extension stage and the regression stage. In this process, the self-repair and progressive injury of the damaged kidney occur almost at the same time. Inhibition and repair of the damaged part and restoration of normal renal function are the key indicators used to evaluate the potential of renal repair. The balance of the two processes determines the long-term outcome after AKI. Renal repair is defined as the recovery of renal structure and function after AKI, but there are still no accurate criteria to evaluate the degree of repair. Complete renal function repair includes renal perfusion, GFR and tubular function recovery. However, the rapid activation of intrinsic repair after renal injury can also induce maladaptive repair that promotes interstitial fibrosis due to several pathophysiological processes. For example, cell cycle arrest leads to the transformation of renal tubular epithelial cells into phenotypes that promote fibroblast growth and activation [22]. As reported by Yang et al., the G2/M-arrest of tubular epithelial cells could activate c-Jun NH2-terminal kinase (JNK) signaling, which in turn promoted the release of profibrotic cytokines [22]. Furthermore, much research has also proved that renal hypoxia induced by injury is also an important factor inducing interstitial fibrosis [23,24]. The continuous fibrosis after AKI further induces the deterioration of renal inflammation and the decrease in capillary density, which can eventually lead to CKD, and even the occurrence and development of subsequent ESRD [20]. According to this, we believe that exploring the potential pathophysiological mechanisms and biochemical pathways of maladaptive repair and finding potential targeted intervention sites to mitigate maladaptive repair after AKI would be of great significance to the prognosis of AKI.

## 3. Pathophysiological Mechanism of Maladaptive Repair

### 3.1. Damage to Renal Tubular Epithelial Cells

As the main components of the kidney, renal tubules and the tubulointerstitium become the most sensitive parts after renal injury [25]. According to previous studies, tubule proliferation often shows up in the straight segment of the proximal tubule, and most of the injury also occurs in this segment [26]. Studies on the replenishment of damaged epithelial cells after injury have shown that the regeneration and replacement of damaged cells by survived epithelial cells are the main mechanism of post-injury repair [27]. The ability to proliferate and replace damaged cells is a key indicator used to determine the success of renal injury repair [28]. Additionally, effective epithelial cell renewal can enable the kidney to recover from almost all kinds of injury such as ischemic, obstructive and toxic injuries. However, with the deepening of the study, we found that, in the process of proliferation, there were still some epithelial cells stagnated in the G2/M phase of the cell cycle. As a result, the injured kidney changed from normal injury repair to pathological fibrosis [29]. Compared with normal proliferative epithelial cells, epithelial cells with stagnant proliferation produce abundant fibrogenic factors and transcriptional growth factors, such as TGF-β, PDGF, CTGF and VEGF [30,31,32,33]. In addition, damaged renal epithelial cells are also closely related to progressive inflammatory responses. When kidney injury occurs, damaged TECs release various danger-associated molecular patterns (DAMPs) through Toll-like receptors (TLRs), Nod-like receptors and NLRP3 inflammasomes to activate innate immunity [34,35,36], and then this process leads to the production of pro-inflammatory cytokines and chemokines in TECs, the recruitment of inflammatory cell infiltration and the promotion of the progressive inflammatory response [37,38,39]. Therefore, we believe that TECs are not only the victim of renal injury but also a potential driver of AKI to CKD. Injured TECs can directly or indirectly advance the occurrence and development of inflammation and fibrosis through various mechanisms. Therefore, protecting renal tubules from repeated damage and restoring healthy renal tubular function may be the key of the treatment of renal diseases.

### 3.2. Endothelial Injury and Sparse Capillary Density

The renal microvascular system plays a key role in the pathophysiology of AKI [40]. Studies of AKI showed that, after renal injury, capillary density and the endothelium were both damaged to some extent. Although the recovery ability of renal tubules is outstanding, the recovery of blood vessels is very different [41]. Studies have shown that the loss of vascular density after renal injury ranging from 30% to 50%, which may also be one of the key inducing factors leading to late fibrosis [42]. For example, studies showed that vasoconstriction, tissue edema, vascular endothelial cell swelling and capillary disintegration lead to capillary sparse in the model of renal ischemia-reperfusion injury (IRI) [23,43,44]. The consequent decrease in blood flow in the damaged area leads to excessive hypoxia in the renal tubular microenvironment, which eventually induces interstitial fibrosis [23,45,46,47]. In the early stage of AKI, the proximal tubule cells were damaged and the secretion of VEGF-A decreased significantly. Then, the decrease of capillary density, decrease of perfusion and hypoxia occur one after another, which will eventually induce vascular endothelial regeneration disturbance [24,41,48]. Consistent with this, in 2016, Chade et al. reported that exogenous administration of VEGF improved the damage of renal microvascular circulation after unilateral renal artery stenosis in pigs and achieved the effect of inhibiting the progression of fibrosis [49]. The kidney has a high energy demand, and adequate oxygen transport is a necessary condition for the kidney to regulate metabolism [50]. However, during AKI, the damage of renal microcirculation breaks the original balance, and the increase of vascular permeability, the occurrence of interstitial edema and the weakening of recovery ability further leads to the aggravation of hypoxia and oxidative stress, which are hidden danger for the maladaptive repair and the occurrence of renal fibrosis after AKI [51].

### 3.3. The Progress of Inflammation

As a common feature of CKD, chronic inflammation is also a key role for the conversion of AKI to CKD. Tonelli et al. have shown that some inflammatory biomarkers, such as CRP, can also reflect the progression of CKD [21]. Based on previous studies, the infiltration of interstitial immune cells and subsequent interstitial fibrosis occur due to inflammation after AKI. In vivo, during the acute stage of ATN, neutrophils can infiltrate rapidly, and monocytes and lymphocytes migrate one after another, which eventually leads to the rapid development of fibrosis [52,53]. The kidney contains an extensive network of resident mononuclear phagocytes [54] which can make a rapid immune response to injury, allowing inflammatory cells to be recruited to the damaged kidney and leading to further aggravation of an early injury; however, on the other hand, they are indispensable factors for post-injury repair [55]. Dying renal tubular cells activate a group of pattern recognition receptors in the renal parenchyma and interstitium, such as Toll-like receptors (TLR) [56], by releasing intracellular molecules, that is, damage-related molecular patterns. In 2014, Kulkarni et al. showed that blocking TLR4 at the early stage of injury can prevent the progression of ATN and AKI, but blocking TLR4 at the recovery stage can inhibit IL-22 production and damage renal regeneration [57].

### 3.4. Interstitial Fibrosis

Renal fibrosis is the main reason for the transformation of AKI to CKD, but the specific mechanism of its occurrence and development has not been fully elucidated. Studies have indicated that the occurrence of renal fibrosis may be a result of changed tubule-interstitial microenvironment. Specifically, the damaged TECs secrete chemokines and cytokines to induce the recruitment of inflammatory cells, and then the latter release proinflammatory factors and profibrotic factors, which aggravates the progression of renal fibrosis [58]. Recent studies have shown the role of myofibroblasts in the progression of fibrosis. These myofibroblasts can produce various types of ECM such as collagens, fibronectins and elastins, which contributes to fibrosis [59]. In addition, as mentioned earlier, sparse capillaries, hypoxia and cell cycle arrest also promote renal fibrosis to varying degrees. Bonventre JV et al. showed proximal tubular cells arrested in the G2/M stage of the cell cycle after injury release profibrogenic growth factors that are capable of stimulating fibroblast proliferation and collagen production [22]. Meanwhile, based on some recent reports, researchers found that the low level of fatty acid oxidation (FAO) in TECs can also contribute to renal fibrosis [60,61]. Kang et al. showed that healthy renal TECs mainly depend on FAO as their energy source, and, whether in animal models or in patients with renal fibrosis themselves, enzymes and regulators of FAO were reduced in the kidney [62]. The progress from AKI to CKD is closely related to the occurrence of persistent fibrosis. It is of great significance to explore the mechanism of fibrosis, find potential targeted intervention sites and inhibit the progress of fibrosis for the repair of AKI and to avoid the occurrence of CKD.

## 4. Biochemical Pathways Leading to Maladaptive Repair after AKI

### 4.1. The Role of Hypoxia-Inducible Factor HIF

**Figure 1:**
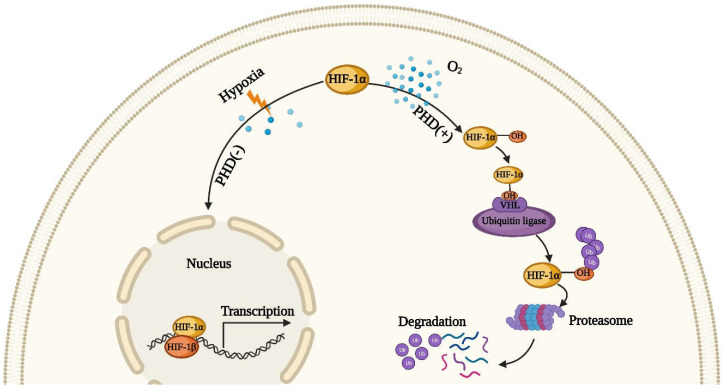
The regulation of hypoxia-inducible factor HIF. In normoxic condition, HIF-1α is hydroxylated by prolyl hydroxylase (PHD), and then hydroxylated HIF-1α can combine with ubiquitin. This compound results in poly-ubiquitination and proteasomal degradation. However, under hypoxia environment, the hydroxylation and hydrolysis of HIF-1α are inhibited, and it binds to HIF-1β to form the HIF-1α/HIF-1β complex in the nucleus, activating the specific gene transcription.

The high metabolic state of the kidney makes its demand for oxygen higher than that of other organs. Because of a greater need for aerobic metabolism, TEC is the most vulnerable cell after mitochondrial dysfunction. After AKI, many factors can lead to hypoxia, such as reductions in capillary density, vasoconstriction and mitochondrial dysfunction. Renal tubules under the hypoxia effect undergo maladaptive repair due to oxidative stress, growth arrest and inhibition of protein synthesis (which is considered to be a known adverse reaction of hypoxia) [63,64,65,66]. Moreover, hypoxia plays a critical role in the subsequent transformation of AKI to CKD, the main reason of which is the activation of hypoxia-inducible factor (HIF) [67]. The activation of HIF is a stress defense mechanism used by cells against a hypoxic microenvironment. HIF, according to the report, is one of the normal heterodimeric helix–loop–helix transcription factors, which is composed of an adjustable oxygen-sensitive α-subunit, HIF-1α and a constitutively expressed β-subunit, HIF-1β [68]. As depicted in Figure 1, in normoxic conditions, HIF-1α is hydroxylated by prolyl hydroxylase (PHD) [69]. The ubiquitin can bond the hydroxylated HIF-1α and then be degraded by the proteasome after the activation of the von Hippel–Lindau tumor suppressor protein (pVHL), with the latter acting as a ubiquitin ligase to advance proteolysis of HIF-1α. When hypoxia occurs, the hydroxylation and hydrolysis of HIF-1α are inhibited, and it binds to HIF-1β to form the HIF-1α/HIF-1β complex in the nucleus, activating the specific gene transcription to regulate cell proliferation [70]. Studies have shown that maintaining the stability of HIF can alleviate the damage of renal cells after AKI [71,72], but this effect is not obvious in TECs [73]. Matsumoto et al. reported that the activation of HIF before AKI induction can relieve the extent of renal injury [74], but it has the opposite effect after AKI induction [75]. Hence, we can see that HIF activation intervention at an appropriate time point after AKI can achieve the best therapeutic effect.

### 4.2. Mitochondrial Dysfunction

As the “energy factory” of the cells, mitochondria is the main power source of kidney cells, especially renal tubular cells [76]. When AKI occurs, TECs are the most easily affected cells by mitochondrial dysfunction because they are more dependent on aerobic metabolism [77]. In kidney, the production of ATP mainly comes from the oxidation of fatty acids (FA) in the mitochondria, while the substrate FA is mainly obtained by extracellular uptake using FA transporter CD36 or by the deacylation of cellular phospholipids under the action of phospholipase A2 (PLA2) [78]. Due to ischemia and hypoxia after AKI, the respiration of mitochondria is inhibited and converts to the glycolysis mode, which greatly reduces the production of ATP [79]. Since ATP is necessary for actin polymerization, depletion of ATP leads to changes in the cytoskeleton of TECs [80], leading to a breakdown of the brush border, loss of cell–cell contact, disruption of barrier function and cell detachment [81]. Hence, the rapid recovery of mitochondrial function and the production of ATP can determine the progression of AKI to some extent, and studies showed that the fusion and fission of mitochondria determine mitochondrial functional recovery. Brooks et al. showed that during cellular stress, mitochondrial fusion was prevented during fission activation, which eventually led to mitochondrial fracture. As a result, the fragmented mitochondria were highly sensitive to Bax insertion activation and transmutation of the outer membrane, and, ultimately, induced apoptosis [82]. In addition, the inhibition of Drp-1, a fission protein that constricts and cleaves mitochondria, can alleviate the progression of mitochondrial damage, apoptosis and renal injury [83]. The same results have also been shown by Morigi M et al. [84]. Therefore, the regulation of mitochondrial dynamics may provide a new opportunity for the treatment of AKI. Furthermore, mitochondrial biogenesis is also a potential feasible therapeutic target for AKI therapy, which is a homeostasis mechanism that replaces damaged mitochondria under basic conditions and is mainly regulated by PGC-1 α [40]. Many studies have indicated that stimulating the expression of PGC-1 α to promote mitochondrial biogenesis increased the number and functions of the mitochondria [85,86,87], thereby reducing oxidative damage and cell death and accelerating the recovery of renal tubular function [88].

### 4.3. DNA Damage

After AKI, DNA damage caused by various stress signals may induce mutation and genomic instability, and the signal pathway network called DNA damage response (DDR) is activated by the cells that undergo DNA damage [89]. The activation of protein kinases in the DDR sensor is followed by the activation of protein kinases (the executor kinases) and subsequent protein phosphorylation to induce cell cycle arrest or final cell death. Because of its effect on the G2/M phase block, DDR is also strongly associated with the occurrence of renal fibrosis [22]. In recent years, the role of DDR in the progression of AKI to CKD has aroused increasing interest. Studies have shown that tumor suppressor protein p53 plays a key role in the activation of DDR [90]. As a major transcription factor, P53 is inactive because of its quick degradation by ubiquitin ligase MDM2 under normal condition. However, once any cellular pressure occurs, MDM2-mediated degradation is inhibited. Consequently, the p53 protein accumulates and can carry out transcriptional activation, which leads to the expression of some cell cycle inhibitors and pro-apoptotic proteins [91,92,93]. Among these, DNA damage is one of the main stress types that activate p53 [94]. Extensive characterization of the signal pathway connecting DNA damage and p53 indicated that a series of Ser/Thr kinases, such as ATR and phosphorylated and stabilized p53 [95,96]. Moreover, Bonventre, J.V. et al. proved that ATR, a sensor of DNA damage, is crucial for mitigating the maladaptive repair and consequent fibrosis after AKI [97]. Furthermore, some studies have also shown that tyrosine kinase c-Abl plays an important role in DNA damage response, and the nuclear input of c-Abl was proved to be necessary for DNA damage-induced apoptosis [98].

### 4.4. Cell Cycle G2/M Arrest

Previous studies have shown that the disorder of the cell cycle could lead to the progression of AKI to CKD. Specifically, G2/M arrest in PTC after AKI leads to the development of maladaptive repair and subsequent fibrosis [99]. The division rate of renal tubular epithelial cells was considered negligible previously [100]. However, when AKI occurs, renal tubular epithelial cells can increase the division rate in a short time to replace necrotic cells [43]. Yang et al. showed that the renewal of epithelial cells promoted the progression of adaptive repair and G2/M-arrested TECs increased in severe damage [22]. Additionally, their results confirmed that the G2/M-arrested epithelial cells activated c-junNH2-terminal kinase (JNK) signal transduction and upregulated the production of fibrogenic cytokines, and the use of JNK inhibitors or P53 inhibitors could obviously alleviate renal fibrosis [22]. In brief, it is believed that the cell cycle G2/M arrest is related to DNA damage and senescence. Studies showed that cells with mutations or deletions of FAN1, a key protein involved in the repair of DNA damage, are more likely to be affected by DNA damage, genomic instability and stagnation at the late G2 stage [101,102]. In addition, IL-8, secreted by senescent cells, binds to CXCR2 and then leads to the G2/M arrest of mitotic cells by activating the NF-κB and p38MAPK/MK2 pathways [103,104,105].

### 4.5. Epigenetic Changes

Epigenetics refers to heritable changes in gene expression through post-translational modification of protein complexes without changing the potential genetic DNA sequence [106]. Recently, researchers have become increasingly interested in the epigenetic changes after AKI. Epigenetic changes are non-negligible in the progress of AKI to CKD, such as DNA methylation, histone acetylation, and non-coding RNA expression [107]. The studies of CKD patients indicate that DNA methylation and histone modification can largely advance the progression of chronic fibrosis [108]. DNA methylation can regulate gene transcription without changing the primary nucleotide sequence. So, whether physiology or pathology, DNA methylation is a non-ignorable intervention target [109]. DNA methylation allows methyl groups to be added to specific sites of DNA such as the fifth carbon of the cytosine ring or to the nitrogen of position 6 in adenine rings by using DNA methyltransferase (DNMTs). Bechtel et al. indicated that the use of the demethylating agent 5’-azacytidine to inhibit the progression of hypermethylation alleviated the progression of fibrosis both in vivo and in vitro [42]. In addition, histone modification is another significant factor in the progression of fibrosis. Histone is a protein, composed of core histones (H2A, H2B, H3 and H4) and connective histones (H1 and H5), that participates in DNA packaging. As histone is positively charged, it can bind to negatively charged DNA by electrostatic interaction and package it into highly concentrated and ordered chromatin structural units, called nucleosomes [110]. Thus far, many ways of histone modification have been studied, such as acetylation, methylation and phosphorylation. Previous studies have revealed that histone deacetylase inhibitors can accelerate recovery after injury and alleviate fibrosis after AKI induced by ischemia reperfusion and aristolochic acid nephrotoxicity [111,112].

## 5. Potential Intervention Strategies

### 5.1. Intervention Strategies for Hypoxia and Oxidative Stress

Hypoxia induced by sparse capillaries and oxidative stress is an important driving force for fibrosis during the progression of AKI to CKD. Therefore, intervention strategies targeting hypoxia or oxidative stress are significant treatments that can be used to alleviate fibrosis and further slowdown the progression of AKI to CKD. When hypoxia occurs, cells construct their defense system by expressing hypoxia-inducible factor (HIF, the main transcription factor under hypoxia) and Nrf2. Thus, an appropriate intervention may accelerate the secretion of these factors and exert their anti-hypoxia effect. Makiko et al. showed that the injury caused by hypoxia after AKI was alleviated significantly by pre-activating the HIF [74]. Similarly, Kapitsinou PP et al. also reported that the pharmacological inhibition of prolyl hydroxylation of HIF before AKI can relieve fibrosis and targeting the PHD/HIF pathway before ischemia can effectively prevent the progression of AKI to CKD [72]. Moreover, Liu M et al. showed that the upregulation of antioxidant genes through drug-activated Nrf2 can relieve injuries in mice after AKI [113]. As is well known, continuous oxidative stress is a crucial element in the development of fibrosis after AKI. Thus, in theory, inhibiting oxidative stress and upregulating the expression of antioxidant genes is of great significance to prevent the progress of AKI to CKD. Although some application of antioxidant therapy to human research has had limited success, the use of these drugs, such as vitamin C, ascorbate and selenium, does inhibit the progression of AKI to a certain extent and alleviate the systemic harm induced by AKI [114].

### 5.2. Intervention Strategies for Cell Cycle Arrest

As mentioned above, during the progression of AKI to CKD, one of the vital pathological mechanisms is G2/M arrest in the cell cycle of TECs, so interventions targeting G2/M cycle arrest are necessary and significant. In general, the possible intervention methods for G2/M cycle arrest include: (1) approaches such as blocking the ATM pathway preventing cells from activating Chks, a downstream factor that can induce G2/M arrest; (2) overcoming the G2/M checkpoint in cell cycle through some intervention strategies, such as p53 inhibitors and histone deacetylase inhibitors; (3) accelerating the apoptosis of senescent cells; (4) inhibiting the secretion of fibrogenic cytokines and thus leaving related cells in an “unresponsive” state without signal reception; and (5) selective scavenging of senescent cells. Baker et al. reported that INKATTAC, a novel transgene, could selectively clear p16INK4a-postive cells in stagnant cell cycle upon drug treatment [115,116]. Moreover, previous studies in our group have proved that murine double minute 2 (MDM2) can induce cell cycle G2/M arrest and accelerate the progression of AKI to CKD; thus, the intervention targeting MDM2 molecules to inhibit G2/M cycle arrest is also a feasible potential method [117].

### 5.3. Targeted Therapy for Epigenetic Changes

Recently, the effect of epigenetic changes has been regarded as a key factor in the progression of AKI to CKD. Previous studies showed that epigenetic changes, including DNA methylation, histone modification, non-coding RNA and chromatin conformational changes are induced after AKI [118]. It has been reported that the use of HDACi, which can increase histone acetylation, can relieve the injury of AKI and contribute to recovery. Additionally, the use of DNA methylation inhibitors (such as 5′-azacytidine and 5-aza-2′-deoxycytidine) or DNA demethylation activators (such as hydralazine) can effectively alleviate renal interstitial fibrosis after ischemia-reperfusion injury [110]. Additionally, there are some reports indicating that the use of HDAC inhibitors, such as TSA, PTBA and their analogues (UPHD25 and 186), can alleviate post-AKI renal injury in mice, accelerate the recovery of renal function and prevent the transition from AKI to CKD [112,119,120]. However, although the targeted epigenetic change interventions are of great significance to prevent the transition from AKI to CKD, there are some issues worth exploring. For example, the high non-specificity of epigenetic drugs may affect global epigenetic changes. Technological advances in recent years have made epigenetic changes in specific genes possible. Xu X et al. showed that the fusion of endonuclease inactivated high-fidelity Cas9 (dHFCas9) and the TET3 catalytic domain (TET3CD), using the CRISPR/Cas9 technique, could target the demethylation of specific genes under the guidance of RNA (sgRNA) [121]. Therefore, the emergence of such technique can provide a new possibility for targeted epigenetic modification.

### 5.4. The Potential Prospect of Stem Cell Therapy

Mesenchymal stem cells (MSCs) are a kind of pluripotent fibroblast-like cells, which can be isolated from bone marrow, adipose tissue, the umbilical cord and other tissues [122]. Using appropriate differentiation inducing agent, MSCs can achieve directional differentiation, such as into osteocytes and adipocytes [123]. MSCs are easy to culture and expand, so they have broad prospects for cell therapy and clinical application [124]. Previous studies showed that, after AKI, the injection of exogenous MSCs could relieve the apoptosis of TECs, alleviate oxidative stress caused by ROS and inhibit the proliferation of inflammation cells. Additionally, the secretion of a variety of cytokines, chemokines and growth factors secreted by MSCs can also alleviate fibrosis and accelerate the adaptive repair of injured renal tissue [125,126,127,128]. MSCs contribute to the adaptive repair by the regulation of immune system. Studies showed that MSCs can inhibit the occurrence of excessive inflammatory response by interacting with various of immune cells [129,130]. Studies showed that MSCs recruited to the injury site after AKI can reprogram the injured cells and thus motivate their proliferation and promote adaptive repairing through growth factors [131,132]. Preliminary clinical studies have confirmed that allogeneic MSCs have good safety and low immunogenicity in vivo [133,134]. However, numerous clinical trials that can confirm the efficacy of exogenous MSCs in AKI treatment are still limited. Gooch et al. reported that, for patients at high risk of AKI, the injection of exogenous MSCs after cardiac surgery can protect early and late postoperative renal function and prevent the worsening of AKI [135]. Although the application prospect of MSCs is expected, there are still many issues that should be clarified in follow-up research, such as the mechanism of MSC treatment in vivo, the accurate influence of the microenvironment on MSCs, the mode and dose of drug administration and the occurrence of long-term adverse reactions in vivo, and all of these deserve further exploration for MSCs therapy.

### 5.5. Application of Nanotechnology

Due to the existence of physiological protective barriers (such as the mononuclear phagocytic system and the glomerular filtration barrier), it is difficult to achieve effective dose accumulation and targeted drug delivery in kidney in AKI therapy [136]. Therefore, it is of great significance to develop a renal-targeted drug delivery system for the treatment of AKI, which can achieve specific drug delivery to the damaged cells in kidney. Meanwhile, the system can also realize effective drug accumulation in the kidney avoiding the occurrence of systemic adverse reactions is another issue to be addressed. Currently, the development of nanotechnology contributes to the advance of renal-targeted drug delivery systems for AKI therapy. Using nano-carriers such as liposomes, polymer nanoparticles, two-dimensional nanomaterials (such as graphene and black phosphorus) and DNA nanostructures, renal targeted drug delivery can be achieved through specific modifications and drug loading [137,138,139]. In 2020, Wang L et al. reported that intravenous injection of modified black phosphorus nanosheets to AKI patients could clear excessive ROS in injury sites, reduce oxidative stress and inhibit the progression of AKI through their innate antioxidant properties [140]. Similarly, Cai W et al. synthesized DNA nanocomplexes based on the filtration characteristics of the glomerular basement membrane (GBM) in vitro and achieved targeted accumulation of DNA complexes in the kidney within a short time after intravenous injection. Additionally, the antioxidant properties of nucleic acid nanostructures also promoted ROS scavenging after AKI and thus inhibited the subsequent progress of AKI [141]. All of these studies indicate that the application of nanotechnology in the kidney disease therapy has extraordinary prospects. Based on the filtration characteristics of GBM, a reasonable modification and drug loading of the nanocarriers could achieve targeted renal accumulation and specific drug delivery.

## 6. Conclusions and Prospects

**Figure 2:**
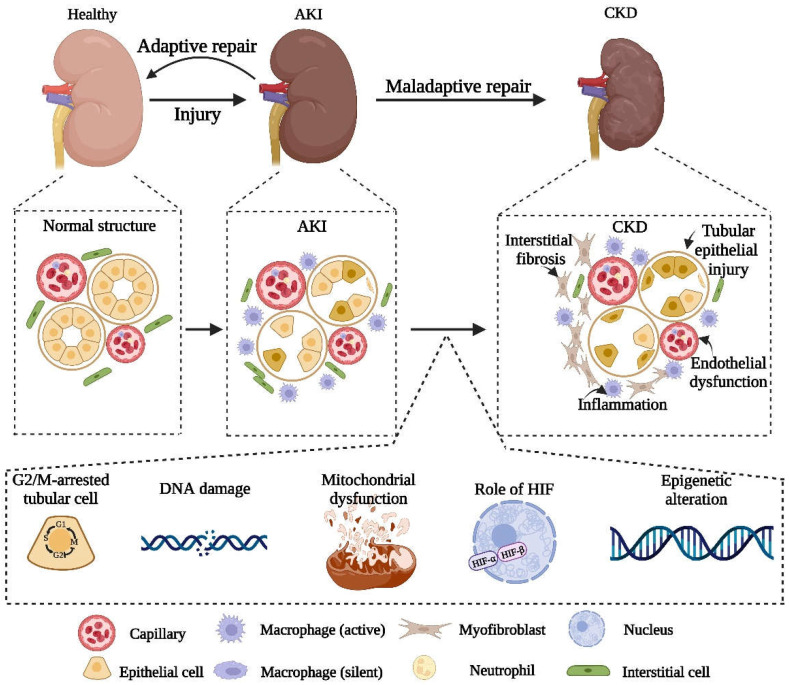
Schematic diagram of pathophysiology and related biochemical pathways in the progression of AKI to CKD.

In summary, there is a close relationship between AKI and CKD. The severity, duration and frequency of AKI are closely related to the possible subsequent CKD progress. AKI itself is a self-healing process, but there is still the possibility of maladaptive repair. If severe renal injury or maladaptive repair occurs, subsequent tubular atrophy, interstitial fibrosis, and renal dysfunction would eventually induce the development of CKD. Accordingly, this review elucidates the mechanisms of maladaptive repair after AKI, including injury of the renal tubular epithelial cells, vascular endothelial damage, sparse capillary density, inflammation and interstitial fibrosis. Additionally, we clarify the possible involved pathways, such as hypoxia, mitochondrial dysfunction, DNA damage and the G2/M cell cycle arrest (Figure 2). Previous studies showed that interventions targeting the related mechanisms could alleviate the fibrosis after AKI and inhibit the occurrence of CKD. In spite of this, there are still some issues to be further explored in the process of AKI to CKD. For example, which is the initial and decisive force of fibrosis after AKI? Is there an obvious causal relationship between these mechanisms? To avoid the progression of AKI to CKD, it is significant to further explore the effective mechanisms in maladaptive repair and seek new possible targets.

## Data Availability

Not applicable.
